# NTD Remodeling in the SARS-CoV-2 BA.3.2 Variant May Influence Spike Stability and Immune Escape

**DOI:** 10.3390/pathogens15070760

**Published:** 2026-07-20

**Authors:** Miriana Quaranta, Alessandra Ciccozzi, Francesco Branda, Leonardo Sernicola, Massimo Ciccozzi, Stefano Pascarella, Alessandra Borsetti, Fabio Scarpa

**Affiliations:** 1 Department of Biochemical Sciences “A. Rossi Fanelli”, Sapienza University of Rome, 00185 Rome, Italy; miriana.quaranta@uniroma1.it (M.Q.); stefano.pascarella@uniroma1.it (S.P.); 2 Sciences and Technologies for Sustainable Development and One Health, Università Campus Bio-Medico di Roma, 00128 Roma, Italy; alessandra.ciccozzi@unicampus.it; 3 Unit of Medical Statistics and Molecular Epidemiology, Università Campus Bio-Medico di Roma, 00128 Rome, Italy; f.branda@unicampus.it (F.B.); m.ciccozzi@unicampus.it (M.C.); 4 National HIV/AIDS Research Center, Istituto Superiore di Sanità, Viale Regina Elena 299, 00161 Rome, Italy; leonardo.sernicola@iss.it; 5 Department of Biomedical Sciences, University of Sassari, Viale San Pietro, 07100 Sassari, Italy; fscarpa@uniss.it

**Keywords:** SARS-CoV-2, cicada, BA.3, BA.3.2, molecular dynamics, free energy landscape, phylodynamics, genetics

## Abstract

In November 2024, a highly mutated descendant of the Omicron BA.3 subvariant, designated BA.3.2, emerged in South Africa carrying 39 spike mutations, two large N-terminal domain (NTD) deletions and a novel four-amino acid insertion. A key feature of BA.3.2 is extensive NTD remodeling, including a major deletion spanning residues 135–148 affecting the β-hairpin region and contributing to the loss of most of the N1 loop. This study compares the evolutionary dynamics and structural features of BA.3.2 with BA.3. Phylodynamic analyses show that BA.3 underwent early demographic stability followed by a decline in genetic diversity, consistent with limited circulation, whereas BA.3.2 displays recent emergence and a progressive reduction in effective population size without rapid expansion. Selection analyses indicate BA.3 evolution is mainly driven by changes in the receptor-binding domain, while BA.3.2 shows dispersed signals across spike regions, including codon 1162. Structural and molecular dynamic analyses reveal increased flexibility and a broader conformational landscape in the BA.3.2 NTD, driven by the deletion and resulting loss of stabilizing interactions. Overall, BA.3.2 follows a distinct evolutionary trajectory characterized by antigenic remodeling of the spike NTD, underlining the need for continued surveillance of emerging SARS-CoV-2 descendant lineages.

## 1. Introduction

Severe Acute Respiratory Syndrome Coronavirus 2 (SARS-CoV-2) evolution has been punctuated by two marked antigenic shifts. The first occurred in late 2021 with the highly divergent Omicron variant (B.1.1.529), characterized by extensive spike remodeling and unprecedented immune escape. A second major shift emerged in July 2023 with the identification of BA.2.86, a highly mutated descendant of BA.2, which rapidly expanded and became globally predominant in 2024. A potential third major evolutionary shift in SARS-CoV-2 has been emerging since late 2024. In November 2024 and January 2025, a highly mutated descendant of the Omicron subvariant BA.3 was identified in South Africa and was designated BA.3.2. The Omicron subvariant BA.3, identified in South Africa in November 2021 in June 2022, disappeared from global circulation [1]. A limited geographic spread and only 760 BA.3 genome sequences were reported worldwide [2], largely confined to southern Africa [3]. No specific mutations were found for the BA.3 lineage in its spike protein, which is a combination of mutations in BA.1 and BA.2 spike proteins. In the spike protein, the N-terminal domain of BA.3 matched that of BA.1, except for the absence of the BA.1-specific EPE insertion. In contrast, all regions downstream of the receptor-binding domain (RBD) were identical to BA.2. The RBD itself, along with the rest of the genome, consisted of a combination of mutations derived from both BA.1 and BA.2 [4]. Its re-emergence in November 2024 marked by identification of the sublineage BA.3.2 in South Africa was unanticipated, and, so far, it has caused relatively few infections, but these are widely distributed, including Europe, Australia, and the United States (covSPECTRUM 2025). Since BA.3.2 contains 39 spike mutations, two large N-terminal domain deletions, and a novel four-residue insertion, relative to the BA.3 subvariant and no known circulating intermediates, it is thought to evolve in persistent infections in immunocompromised people in the southern African region [5]. This region remains the primary site for the emergence of Omicron variants, maybe due to the high prevalence of people with impaired immune function because of Human Immunodeficiency Virus (HIV) disease. Two BA.3.2 S protein sequences designated BA.3.2 (A) and BA.3.2 (B) have been identified. They differ by four mutations, supporting the existence of distinct BA.3.2 subvariants. BA.3.2 underwent extensive remodeling of the spike N-terminal domain (NTD). In addition to the BA.3-inherited deletions (Δ69-70, Δ142-144, and ΔN211), BA.3.2 accumulated eleven further deletions, including a markedly expanded N3-loop deletion (Δ136-147) and a two-residue deletion in the N5 loop (Δ243-244). In addition, the NTDs of BA.3 and BA.3.2 differ by nine amino acid substitutions: P9L, R21T, P26L, I101T, F157S, N164K, S172F, K187T, and P251S [1]. A distinctive feature of the BA.3.2 NTD is the likely loss of most of the N1 loop. Specifically, the P9L substitution shifts the signal peptide cleavage site from residues 13–14 to residues 21–22 [6], thereby potentially resulting in the effective removal of the majority of the N1 loop. The loss of most of the N1 loop, together with the Δ136–147 deletion, results in disruption of the Cys15–Cys136 disulfide bond and may drive structural rearrangement of the NTD, thereby promoting escape from NTD-specific neutralizing antibodies [7,8,9]. Hence, length polymorphisms within the N loops modulate SARS-CoV-2 spike activity. Although variation in loop length can influence neutralization by NTD-targeting monoclonal antibodies (mAbs), immune selection is not required to drive the emergence of N-loop variants. Notably, the functional consequences of loop length alterations appear to be context-dependent, yielding divergent effects depending on the structural and evolutionary background [10]. The pronounced antibody evasion exhibited by BA.3.2 likely arises from a combination of point mutations in the N1 and N5 loops and a deletion within the N3 loop of the NTD antigenic supersite [11]. The substantial spread of BA.3.2.2 in Perth (population ~2 million), together with its detection in Sydney on 3 September 2025 (EPI_ISL_20187886, GISAID), may facilitate the acquisition of additional adaptive mutations by this variant thereby warranting close monitoring. In this context, we carried out an integrated genomic investigation combining genetic diversity, phylodynamic, and structural analyses to comprehensively characterize the epidemiological dynamics and potential public health implications of BA.3 and BA.3.2 descendant lineages. Particular emphasis was placed on identifying distinctive molecular and evolutionary features that may have contributed to the expansion of BA.3 relative to its parental lineages.

## 2. Materials and Methods

To obtain a deep understanding of the molecular evolution of the SARS-CoV-2 BA.3.2 lineage, a comparison of its evolutionary dynamics with that of its progenitor BA.3 was performed. For the analyses all available spike gene sequences assigned to BA.3 and BA.3.2 were retrieved from the GISAID database (https://gisaid.org, accessed on 4 June 2026). See Supplementary File S1 for details on the used genomes. Sequences with incomplete sampling dates, low coverage, or excessive ambiguities were excluded from the analyses. Independent datasets were generated for the BA.3 (*n* = 130, collected between 30 November 2021 and 9 March 2026) and BA.3.2 (*n* = 114, collected between 15 July 2025 and 9 May 2026) lineages. Each dataset was aligned separately using the L-INS-I algorithm implemented in MAFFT (Multiple Alignment using Fast Fourier Transform) v7.471 [12]. Sequence alignments were manually inspected and edited using Unipro UGENE v.35 [13]. Evolutionary rate and changes in viral genetic diversity were co-estimated within a Bayesian framework using BEAST (Bayesian Evolutionary Analysis Sampling Trees) v1.10.4 [14], for both datasets. Markov Chain Monte Carlo (MCMC) analyses were run for 400 million generations under the Bayesian Skyline Plot (BSP) coalescent model implemented in the uncorrelated lognormal clock model, with parameters sampled every 20,000 generations. Bayesian Skyline analyses were performed independently for the BA.3 and BA.3.2 datasets using the software Tracer v1.7 [15], allowing the reconstruction of temporal changes in effective population size (Ne) and comparison of the demographic histories of the two lineages.

To investigate the selective pressures acting on viral coding sequences, codon-based evolutionary analyses were performed using the FUBAR (Fast, Unconstrained Bayesian AppRoximation) [16] and MEME (Mixed Effects Model of Evolution) [17] methods implemented in the software HyPhy v.2.5.101 [18].

Amino acid sequences and N-glycosylation positions of the NTDs from BA.3 and BA.3.2 were extrapolated from data reported in [19].

Three-dimensional structural models of the NTDs were generated and validated by the server SwissModel [20] using the PDB templates 7R18 and 8THF, respectively. N-glycosylation was reproduced on the structural models using the tools available on the CHARMM-GUI server [21]. A single unit of N-acetyl-glucosamine (NAG) was connected to the Asn residues of each N-glycosylation site.

The net charge of each domain was calculated with the program PROPKA3 [22].

To simplify the molecular system, molecular dynamics simulations were carried out using the three-dimensional models of the NTDs without NAG units. Calculations were performed using the GROMACS v.2024 program [23]. The NTD molecules were placed in a dodecahedral box, solvated with TIP3P water molecules, and positioned at a 1.5 nm distance to the box edge. The solvated system was neutralized to a final concentration of 0.15 M NaCl. The system was energy-minimized using the steepest descent algorithm until the maximum force was less than 1000.0 kJ/mol/nm. The system underwent 10 ns of NVT equilibration followed by 10 ns of NPT equilibration at 300 K with a modified Berendsen thermostat (time constant 1 ps). The time step was set to 2 fs. The production phase of the simulations was conducted in the NPT ensemble. The LINCS algorithm [24] was applied to constrain the bond lengths. Electrostatic forces were calculated with the Particle Mesh Ewald method [25] using a grid spacing of 0.16 nm. A cutoff of 1.0 nm was set for short-range electrostatic and van der Waals interactions. All simulations were conducted for 500 ns in triplicate. The time step was increased to 4 fs after application of hydrogen mass repartitioning [26].

Trajectories were visualized with the software VMD [27] and analyzed with the GROMACS tools combined with the XMGRACE plotting program [28]. Molecular structures were displayed with the graphic programs PyMOL [29] or ChimeraX [30]. Potential energy, Root Mean Square Deviation (RMSD), Root Mean Square Fluctuation (RMSF), and Radius of Gyration were calculated with the GROMACS tools to monitor the stability of the systems in each triplicate simulation.

The conformations adopted by the BA.3 and BA.3.2 NTDs during the simulations were clustered using the gmx cluster function in GROMACS, with the gromos clustering algorithm [31]. The conformational space explored by the BA.3 and BA.3.2 N-terminal domains (NTDs) during molecular dynamics simulations was analyzed using principal component analysis (PCA) to identify the main collective motions of the system. A free energy landscape (FEL) was reconstructed based on the distribution of conformations in the first two principal components’ PC1-PC2 space. The free energy surface was obtained using GROMACS tools, and the resulting data were interpolated on a regular grid using the SciPy Python library v. 1.16 and visualized as three-dimensional energy surfaces using Matplotlib v. 3.10.

## 3. Results

### 3.1. Phylodynamics

Phylodynamic analyses were performed on the spike gene. The evolutionary rate estimated for BA.3 was 1.679 × 10^−3^ substitutions/site/year (95% HPD: 8.08 × 10^−4^–2.79 × 10^−3^), whereas BA.3.2 showed a slightly higher mean evolutionary rate of 2.211 × 10^−3^ substitutions/site/year (95% HPD: 9.9 × 10^−4^–3.6 × 10^−3^). The estimated root age was 2020.9 for BA.3 and 2023.99 for BA.3.2.

**Figure 1:**
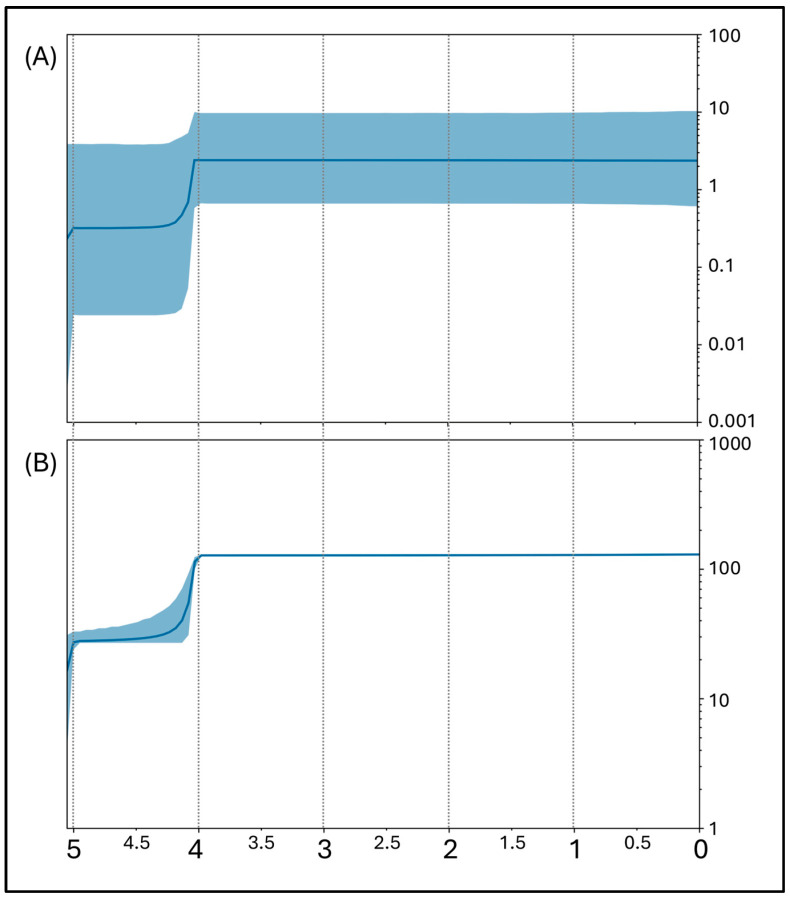
Bayesian Skyline Plot (BSP; (**A**) and lineages through time (LTT; (**B**) analyses inferred from the dataset of the BA.3 lineage. In panel (**A**), the solid blue line represents the median estimate of effective population size (Ne), while the shaded area indicates the 95% Highest Posterior Density (HPD) interval. In panel (**B**), the LTT curve depicts the cumulative accumulation of lineages through time and shows a corresponding increase in lineage diversification during the same period. Time is expressed in years before the most recent sample (9 March 2026; t = 0).

The Bayesian Skyline Plot (BSP) reconstruction of BA.3 (Figure 1A) revealed a prolonged phase of demographic stability. The median effective population size (Ne) remained nearly constant from the estimated origin of the lineage until approximately 4.0 years before the most recent sampling date (9 March 2026), corresponding to March–April 2022. Subsequently, a marked contraction in relative genetic diversity was observed between approximately 4.2 and 4.0 years before 9 March 2026, corresponding approximately to the period between late 2021 and spring 2022. Following this decline, the skyline reached a second plateau characterized by substantially lower Ne values, which persisted until the most recent sampling date. The 95% HPD interval remained relatively broad throughout the reconstruction but supported an overall pattern of rapid reduction in Ne followed by persistence at lower levels.

Likewise, the lineage through time (LTT) reconstruction (Figure 1B) revealed an extended phase characterized by a relatively constant number of lineages, which persisted until around 4.0 years before 9 March 2026 (March–April 2022). A rapid decline in the number of lineages was then observed. Thereafter, the number of lineages remained relatively stable until the most recent sampling date. Overall, the pattern indicates an initial diversification phase followed by a marked reduction in phylogenetic diversity.

**Figure 2:**
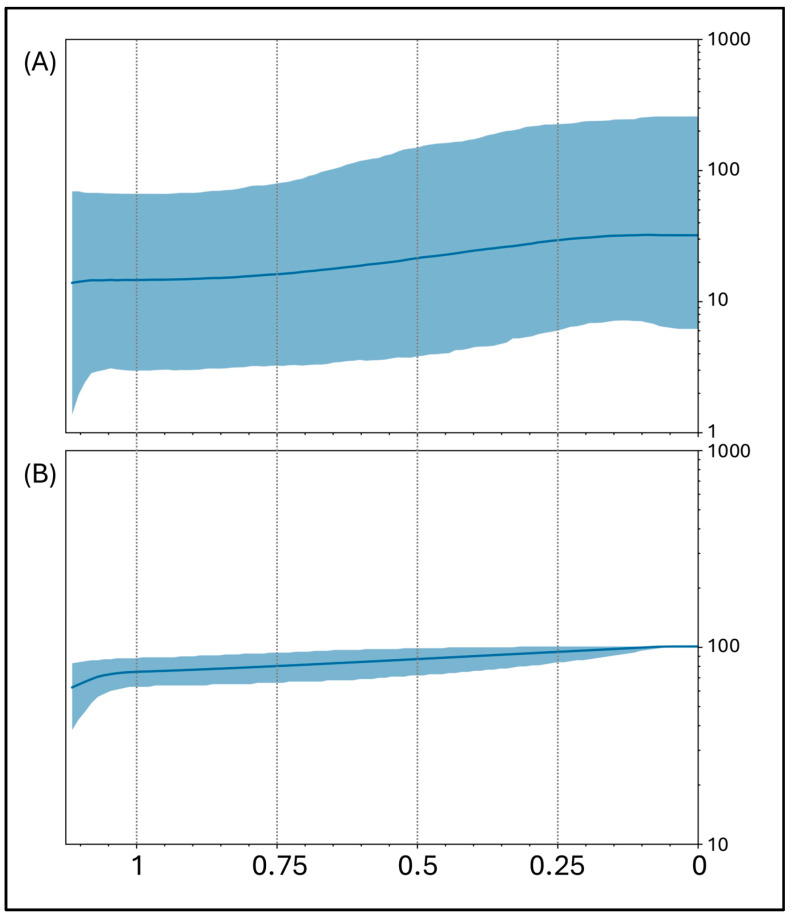
Bayesian Skyline Plot (BSP; (**A**) and lineages through time (LTT; (**B**) analyses inferred from the dataset of the BA.3.2 lineage. In panel (**A**), the solid blue line represents the median estimate of effective population size (Ne), while the shaded area indicates the 95% Highest Posterior Density (HPD) interval. In panel (**B**), the LTT curve depicts the cumulative accumulation of lineages through time and shows a corresponding increase in lineage diversification during the same period. Time is expressed in years before the most recent sample (9 May 2026; t = 0).

The BSP reconstruction of BA.3.2 (Figure 2A) covered a substantially shorter temporal interval. The median effective population size was highest close to the estimated origin of the lineage and gradually decreased through time. At approximately 1.1–1.2 years before the most recent sampling date (9 May 2026), corresponding to February–March 2025, the highest median Ne values were observed. No evidence of abrupt demographic expansion was detected during the reconstructed history. Instead, the skyline showed a progressive decline in relative genetic diversity toward the most recent sampling date. The 95% HPD interval remained broad throughout the analysis, although the overall decreasing trend consistently recovered. The LTT analysis (Figure 2B) showed that, beginning approximately 1.1–1.2 years before 9 May 2026 (February–March 2025), the number of lineages gradually decreased over time without evidence of rapid diversification events. The reduction became more evident approaching the most recent sampling date, when the number of lineages declined substantially. Overall, the lineage accumulation pattern suggests limited diversification and a progressive reduction in ancestral lineages throughout the evolutionary history of BA.3.2.

For the dataset of BA.3, FUBAR identified pervasive positive selection at codons 67, 95, 339, 371, 405, 417, 440, 446, 452, 477, 478, 484, 498, 501, 505, 681, and 852 (Table 1). MEME detected episodic diversifying selection at codons 95, 501, 505, and 700 (Table 1). Notably, codons 95, 501, and 505 were supported by both methods, indicating robust evidence of adaptive evolution at these sites. Most positively selected codons were concentrated within the receptor-binding domain (RBD), particularly in the receptor-binding motif (RBM), highlighting strong selective pressures acting on regions involved in host receptor interaction. Additional signals of positive selection were detected in the N-terminal domain (NTD), the S1/S2 cleavage site region, and the S2 subunit.

Notably, the majority of positively selected codons were concentrated within the RBD, particularly in the RBM. Several of these sites (e.g., 417, 440, 446, 452, 477, 478, 484, 498, 501, and 505) have previously been implicated in antigenic evolution and ACE2 receptor interaction, suggesting that adaptive evolution in the BA.3 dataset primarily targeted functionally important regions involved in host adaptation and immune escape.

For the dataset of BA.3.2, FUBAR identified seven codons under pervasive positive selection (172, 326, 339, 440, 679, 852, and 1162) and one codon under pervasive purifying selection (340) (Table 2). MEME detected episodic diversifying selection at codons 214, 356, and 1162 (Table 2). Codon 1162 was supported by both methods and therefore represents the strongest candidate for adaptive evolution in the dataset.

### 3.2. Sequence and Structure Comparison

**Figure 3:**
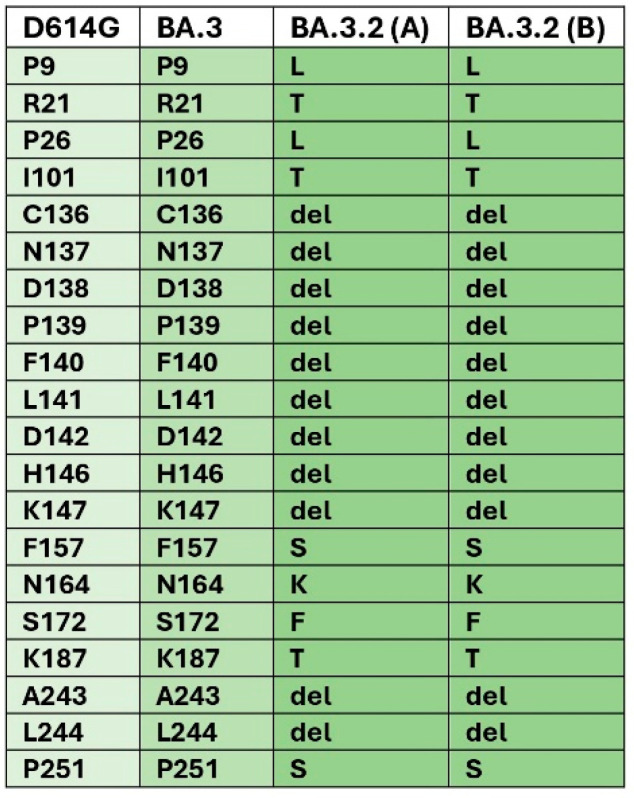
Comparison of NTD mutations in the BA.3, BA.3.2 (A), and BA.3.2 (B) variants relative to wild type (D614G).

**Figure 4:**
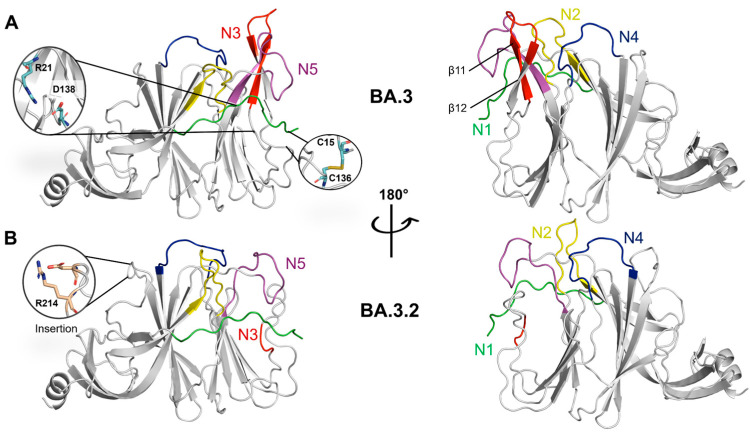
Structural representation of the homology models of the BA.3 (**A**) and BA.3.2 (**B**) NTDs. Both variants are shown from one side and rotated by 180°. The N1 (green), N2 (yellow), N3 (red), N4 (blue), and N5 (magenta) loops are labeled in both variants. In BA.3 (**A**), a close-up of the region containing the disulfide bridge between Cys15 and Cys136 and the salt bridge between Arg21 and Asp138 is shown, with the residues represented as sticks and labeled. In BA.3.2 (**B**), a close-up of the region containing the insertion and the potential salt bridge between the inserted Asp and Arg214 is shown with the residues represented as sticks and labeled.

Figure 3 and Figure 4 report the comparison of the mutations characterizing the wild type, BA.3 and the BA.3.2 variants and the structural representation of the homology models of the BA.3 and BA.3.2 NTDs, respectively. Supplementary Figure S1 displays the sequence comparison among the reference, BA.3 and BA.3.2 NTDs. The sequence of BA.3.2 is characterized by a large deletion encompassed by sequence positions 135–148 (numbering refers to the complete wild-type spike sequence). The deletions obliterate one *β*-strand of the *β*-hairpin in positions 144–157 formed by the strands *β*11 and *β*12, resulting in a massive local rearrangement of the conformation of the loop N3. The deletion removes the disulfide bridge between Cys15 and Cys136 and the salt bridge between Arg21 and Asp138 that are present in BA.3. Moreover, the structural rearrangement moves the glycosylated Asn134 from the loop of the *β*-hairpin as in BA.3, near the glycosylated Asn17 at the N-terminus region of the domain. The conformation of loop N3 appears to be the most affected by the deletion, while the other loops N1, N2, N5 and N5 are conserved in the two variants.

The insertion of the sequence “ASDT” occurring in BA.3.2 within the loop at sequence positions 213 and 214 introduces a potential salt bridge between the inserted Asp and Arg214.

The net charges of the BA.3 and BA.3.2 NTDs calculated with the program PROPKA3 were 0.13 and −0.79, respectively. Supplementary Figure S2 shows the surface electrostatic potentials of BA.3 and BA.3.2.

### 3.3. Molecular Dynamics

**Figure 5:**
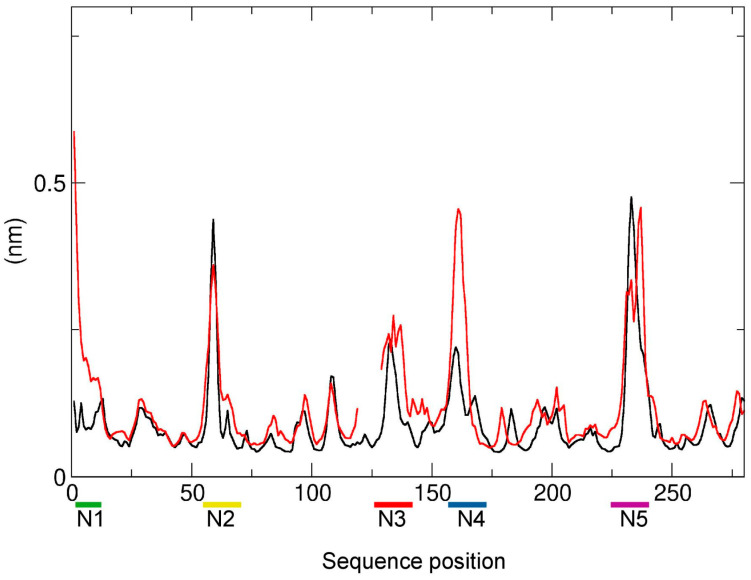
Backbone RMSF of BA.3 (black line) and BA.3.2 (red line). The two plots have been aligned according to the sequence alignment reported in Supplementary Figure S1. Position of the N loops is indicated by lines colored as in Figure 4.

The molecular dynamics of the two NTD domains was simulated for 500 ns in triplicate after energy minimization and equilibration. Calculation of RMSD and radius of gyration suggests that the two domains are stable over the entire simulation (Supplementary Figures S3–S5). Backbone RMSF was calculated to identify differences in fluctuation of the local conformation. The most evident difference lies in the high flexibility of the N-terminal segment of the BA.3.2 NTD (Figure 5) consisting of approximately the first 10 amino acids. The increased flexibility is a consequence of the loss of the disulfide bridge between the Cys15 and Cys136 and the salt bridge between Arg21 and Asp138. Removal of these interactions loosens the contact between the N-terminus segment and the surrounding structural environment. Moreover, loop N4 (sequence position 177–187) displays an increased flexibility (Figure 5) that appears to be a consequence of the *β*-hairpin deletion and of the loss of interactions between the two portions of the domain. Interestingly, the loop is in proximity to the glycosylation site at Asn99, which is absent in BA.3. The other unique BA.3.2 glycosylation site is at Asn185, located in the exposed loop connecting the *β*14 and *β*15 strands (Supplementary Figure S6).

Free energy landscape (FEL) analysis (Supplementary Figure S7) shows that the NTD of BA.3.2 explores a conformational space wider than that of BA.3 and with delocalized local energy minima.

## 4. Discussion

Omicron continues to diversify despite increasing population immunity. Most emerging sublineages acquire Spike substitutions, deletions, and insertions that incrementally enhance viral fitness and transmissibility, the latest being the heavily mutated BA.3.2. Genetic analyses overall, indicated that BA.3 and BA.3.2 displayed comparable evolutionary rates but markedly different demographic histories, with BA.3.2 representing a recently emerged lineage showing limited diversification. Phylodynamic analyses indicate that, despite its recent emergence and extensive Spike remodeling, BA.3.2 showed no evidence of rapid demographic expansion. This suggests that the marked structural changes accumulated in the Spike protein were not accompanied by a corresponding increase in genetic diversity at the population level.

The combination of a recent origin, extensive spike remodeling, and the absence of known evolutionary intermediates is compatible with the hypothesis that BA.3.2 may have evolved in a poorly sampled reservoir, potentially during prolonged viral replication. Similar evolutionary patterns have been proposed for other highly divergent SARS-CoV-2 lineages characterized by long phylogenetic branches and the rapid accumulation of multiple spike mutations. Although the present analyses do not directly address the mechanism underlying the emergence of BA.3.2, the phylodynamic results support the view that the lineage followed an evolutionary trajectory distinct from that of its BA.3 ancestor.

Importantly, our structural analyses show that many defining BA.3.2 mutations converge on the NTD antigenic supersite. The extensive β-hairpin deletion, disruption of the C15–C136 disulfide bond, increased N-terminal flexibility, and broader conformational landscape collectively indicate substantial antigenic remodeling. Selection analyses revealed distinct evolutionary patterns between the BA.3 and BA.3.2 datasets. In BA.3, signatures of positive selection were predominantly concentrated within the receptor-binding domain (RBD), particularly in the receptor-binding motif (RBM), indicating that adaptive evolution mainly targeted regions directly involved in host receptor recognition and immune escape.

In contrast, the BA.3.2 dataset exhibited fewer positively selected sites distributed across multiple Spike domains rather than being concentrated within the RBM. The identification of codon 1162 by both FUBAR and MEME suggests that adaptive evolution in BA.3.2 may also involve structural elements outside the receptor-binding region.

Overall, these findings suggest that the evolutionary trajectories of BA.3 and BA.3.2 may have been shaped by different selective landscapes. While BA.3 appears to have undergone extensive adaptive evolution in immune-relevant and receptor-binding regions, BA.3.2 displays a more dispersed pattern of selection, potentially reflecting distinct functional constraints and adaptive strategies during its diversification.

The defining feature of the BA.3.2 NTD is a nine-residue deletion in region 136-147, evaluated via in silico structural analyses [32,33]. This deletion removes most of the β-hairpin (β11 and β12 strands), disrupting the conformation of the residual N3 loop and destabilizing the N-terminal portion of the domain.

The N3 loop is a key component of the antigenic supersite targeted by several monoclonal antibodies (mAbs) [34,35], where even minimal changes like ΔY144 trigger antibody escape [36]. Furthermore, loop alterations similar to N5-loop deletions driving N4-loop rearrangements [37] or those reported in the Lambda variant [38] strongly affect mAb recognition. In BA.3.2, the deletion of the N3 loop directly increases the flexibility of the adjacent N4 loop, forcing the entire domain to sample a wider conformational space than that of BA.3.

Increased flexibility of antigenic loops impairs antibody recognition by destabilizing the native epitope conformation required for binding [39], which relies on amino acid composition, structural integrity, and conformational stability [34]. Consequently, the disruption of the Cys15–Cys136 disulfide bond, the remodeling of the N3 supersite, and the heightened mobility of adjacent loops collectively lower the binding probability of antibodies targeting the native NTD supersite [40]. This underscores why NTD-directed mAbs may show reduced efficacy across different variants.

Regarding electrostatic potential, which heavily dictates molecular interactions [41], recent SARS-CoV-2 variants have progressively shifted toward more-negative net charges [42]. BA.3 and BA.3.2 deviate from this trend: BA.3 exhibits an almost neutral net charge due to a localized positive surface patch that neutralizes surrounding negative charges, while BA.3.2 lacks this positive patch, resulting in a moderately negative charge of −0.79 similar to HV.1, which belongs to the XBB lineage, (−0.78) but less negative than BA.2.86 (−1.72).

Free energy landscape (FEL) comparisons reveal distinct behaviors: BA.3 displays a localized, stable low-energy basin with limited states, whereas BA.3.2 exhibits a broader, more-rugged landscape with multiple local minima. This dynamic heterogeneity and flexibility are driven by the NTD deletion, allowing BA.3.2 to explore alternative structural substates. Lastly, because the NTD modulates both viral entry and spike fusogenicity [43], this extensive structural remodeling likely explains why BA.3.2 enters cells slightly less efficiently than its parental BA.3 variant.

It is worth emphasizing that, although O-glycosylation is less extensive than N-glycosylation [44,45], accumulating evidence suggests that it may influence the structural and antigenic properties of the SARS-CoV-2 Spike protein. In particular, O-glycans located in exposed regions, including NTD and the S1/S2 cleavage site, have been proposed to modulate protein conformation, protease processing, and immune recognition [46,47]. However, O-glycosylation sites within the NTD remain difficult to predict and accurately characterize experimentally. Accordingly, the present study was designed to investigate N-glycosylation exclusively, without addressing O-glycosylation.

To the best of our knowledge, this is the first report to combine molecular modeling and simulation techniques to characterize the structural consequences of this large deletion in the Spike NTD.

## 5. Conclusions

This study provides an integrated phylodynamic, evolutionary, and structural characterization of the SARS-CoV-2 BA.3 and BA.3.2 lineages, highlighting marked differences in their evolutionary trajectories. While BA.3 shows a pattern consistent with limited circulation followed by a reduction in genetic diversity, BA.3.2 emerges as a recently evolved lineage with a distinct and more-complex evolutionary profile, despite the absence of a clear demographic expansion. The two lineages are shaped by different selective landscapes, with BA.3 primarily driven by adaptive changes in the receptor-binding domain, whereas BA.3.2 displays more-dispersed selective pressures across multiple regions. Structural and molecular dynamics analyses further indicate that BA.3.2 has undergone a high remodeling of the NTD, driven by extensive deletions and impairment of interactions, resulting in increased conformational flexibility and altered electrostatic properties. All these findings suggest that BA.3.2 follows an evolutionary pathway distinct from its ancestor, characterized by putative antigenic remodeling of the Spike protein. This supports the need for continued genomic and structural surveillance of emerging SARS-CoV-2 descendant lineages to better understand their evolutionary direction.

## Figures and Tables

**Table 1:** Codon sites inferred to be under positive selection in the Spike protein of BA.3 lineages. Sites identified under pervasive positive selection, episodic diversifying selection, or both are reported together with their corresponding Spike functional domains. Most positively selected codons were located within the receptor-binding domain (RBD), particularly in the receptor-binding motif (RBM).

Reference Codon	Selection Type	Method	Spike Domain
67	Positive selection	FUBAR	NTD
95	Positive + Episodic positive	FUBAR + MEME	NTD
339	Positive selection	FUBAR	RBD
371	Positive selection	FUBAR	RBD
405	Positive selection	FUBAR	RBD
417	Positive selection	FUBAR	RBD
440	Positive selection	FUBAR	RBM
446	Positive selection	FUBAR	RBM
452	Positive selection	FUBAR	RBM
477	Positive selection	FUBAR	RBM
478	Positive selection	FUBAR	RBM
484	Positive selection	FUBAR	RBM
498	Positive selection	FUBAR	RBM
501	Positive + Episodic positive	FUBAR + MEME	RBM
505	Positive + Episodic positive	FUBAR + MEME	RBM
681	Positive selection	FUBAR	S1/S2 cleavage site region
700	Episodic positive selection	MEME	S2
852	Positive selection	FUBAR	S2

**Table 2:** Codon sites inferred to be under positive selection in the Spike protein of BA.3.2 lineages. Sites identified under pervasive positive selection, episodic diversifying selection, or both are reported together with their corresponding Spike functional domains. Most positively selected codons were located within the receptor-binding domain (RBD), particularly in the receptor-binding motif (RBM).

Reference Codon	Selection Type	Method	Spike Domain/Region
172	Positive selection	FUBAR	NTD
214	Episodic positive selection	MEME	NTD
326	Positive selection	FUBAR	RBD
339	Positive selection	FUBAR	RBD
340	Purifying (negative) selection	FUBAR	RBD
356	Episodic positive selection	MEME	RBD
440	Positive selection	FUBAR	RBM
679	Positive selection	FUBAR	S1/S2 cleavage site region
852	Positive selection	FUBAR	S2 subunit
1162	Positive selection + Episodic positive selection	FUBAR + MEME	HR2 region (Heptad Repeat 2)

## Data Availability

The original contributions presented in this study are included in the article. Further inquiries can be directed to the corresponding author.

## Supplementary Materials

The following supporting information can be downloaded at https://www.mdpi.com/article/10.3390/pathogens15070760/s1: Figure S1: Sequence alignment of the NTDs of the spikes from the reference strain (SPIKE_SARS2), and from BA.3 and BA.3.2 variants; Figure S2: BA.3 and BA.3.2 electrostatic potential surface visualization obtained with PyMOL APBS Electrostatics Plugin; Figure S3: RMSD of the backbone of BA.3.2 NTD in the three replicas of molecular dynamics simulation; Figure S4: RMSD of the backbone of BA.3 NTD in the three replicas of molecular dynamics simulation; Figure S5: Radius of gyration of the backbone of BA.3 and BA.3.2 NTD in the three replicas of molecular dynamics simulation; Figure S6: Map of the N-glycosylation sites on the surface of BA.3 (cyan, on the left) and BA.3.2 (orange, on the right); Figure S7: Free energy landscapes (FELs) of the BA.3 and BA.3.2 variants reconstructed from molecular dynamics trajectories projected onto the first two principal components obtained by principal component analysis (PCA). Supplementary File S1: Genomic sequences used for genetic analyses retrieved from the GISAID database (accessed 4 June 2026). All sequences were publicly available and not subject to embargo restrictions at the time of retrieval. Detailed metadata are provided in Supplementary File S1. The datasets analyzed in this study are available at the following DOIs: https://doi.org/10.55876/gis8.260624eg (SARS-CoV-2 BA.3 lineage) and https://doi.org/10.55876/gis8.260624cq (SARS-CoV-2 BA.3.2 lineage).

## Abbreviations

The following abbreviations are used in this manuscript:

FEL	Free Energy Landscape
GISAID	Global Initiative on Sharing All Influenza Data
MAFFT	Multiple Alignment using Fast Fourier Transform
NTD	N-Terminal Domain
