# Supplementary material for: NTD Remodeling in the SARS-CoV-2 BA.3.2 Variant May Influence Spike Stability and Immune Escape
Source: Pathogens. 2026 Jul 20;15(7):760. doi: 10.3390/pathogens15070760 (PMC13414510; doi:10.3390/pathogens15070760)
Supplement: Supplementary file 1 [file pathogens-15-00760-s001.zip › pathogens-4431490-supplementary-file S1.pdf]

# Supplementary Appendix

All genome sequences and associated metadata supporting the findings of this study can be accessed through the persistent digital object identifier https://doi.org/10.55876/gis8.260624eg

In addition to the minted DOI, GISAID also communicates the aggregation of GISAID accession numbers (EPI_ISL_IDs) through the corresponding EPI_SET_260624eg identifier to facilitate both, the acknowledgment of all data contributors and the direct retrieval of the underlying data from GISAID used in this study.

## hCoV-19 Virus Data Summary

| GISAID Identifier | Digital Object Identifier | Number of individual viruses | Data Collection range | Number of countries/territories |
|---|---|---|---|---|
| EPI_SET_260624eg | https://doi.org/10.55876/gis8.260624eg | 129 | 2021-11-30 to 2026-03-09 | 13 |

# Supplementary Appendix

All genome sequences and associated metadata supporting the findings of this study can be accessed through the persistent digital object identifier https://doi.org/10.55876/gis8.260624cq

In addition to the minted DOI, GISAID also communicates the aggregation of GISAID accession numbers (EPI_ISL_IDs) through the corresponding EPI_SET_260624cq identifier to facilitate both, the acknowledgment of all data contributors and the direct retrieval of the underlying data from GISAID used in this study.

## hCoV-19 Virus Data Summary

| GISAID Identifier | Digital Object Identifier | Number of individual viruses | Data Collection range | Number of countries/territories |
|---|---|---|---|---|
| EPI_SET_260624cq | https://doi.org/10.55876/gis8.260624cq | 115 | 2025-07-15 to 2026-05-09 | 14 |
