# Supplementary material for: NTD Remodeling in the SARS-CoV-2 BA.3.2 Variant May Influence Spike Stability and Immune Escape
Source: Pathogens. 2026 Jul 20;15(7):760. doi: 10.3390/pathogens15070760 (PMC13414510; doi:10.3390/pathogens15070760)
Supplement: Supplementary file 1 [file pathogens-15-00760-s001.zip › pathogens-4431490-supplementary.pptx]

## Slide 1
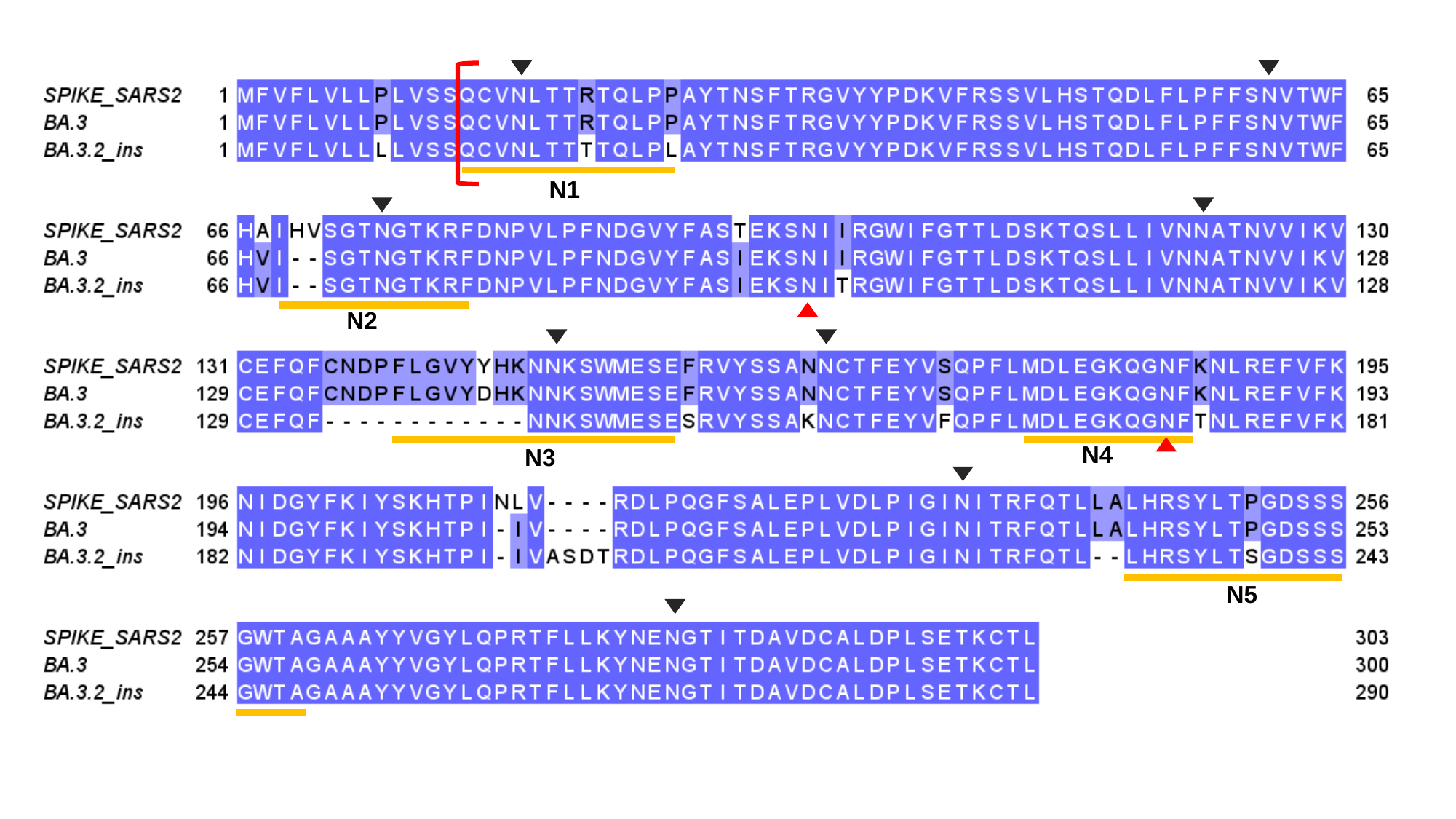

N1
N2
N4
N3
N5

## Slide 2
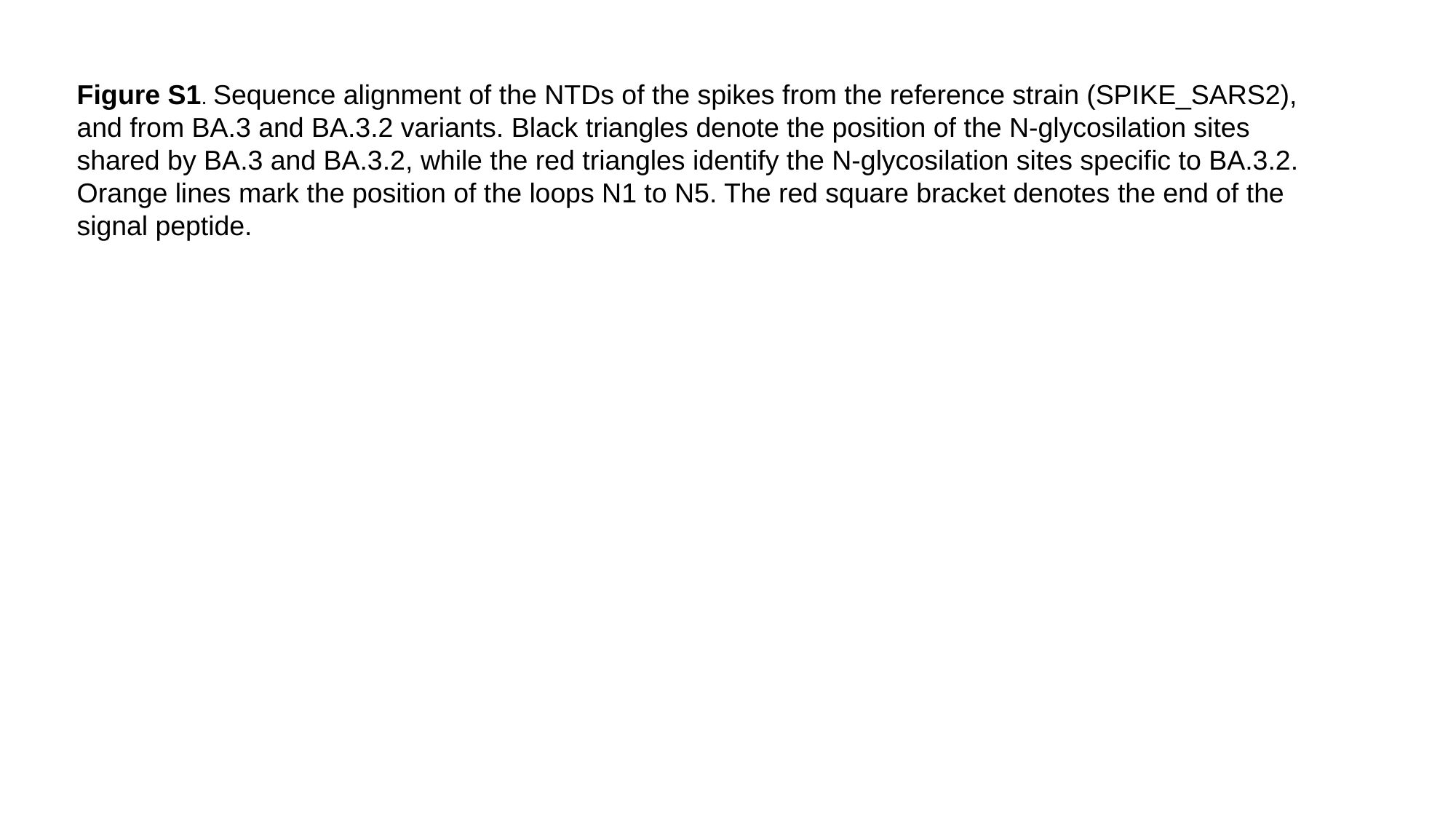

Figure S1. Sequence alignment of the NTDs of the spikes from the reference strain (SPIKE_SARS2), and from BA.3 and BA.3.2 variants. Black triangles denote the position of the N-glycosilation sites shared by BA.3 and BA.3.2, while the red triangles identify the N-glycosilation sites specific to BA.3.2. Orange lines mark the position of the loops N1 to N5. The red square bracket denotes the end of the signal peptide.

## Slide 3
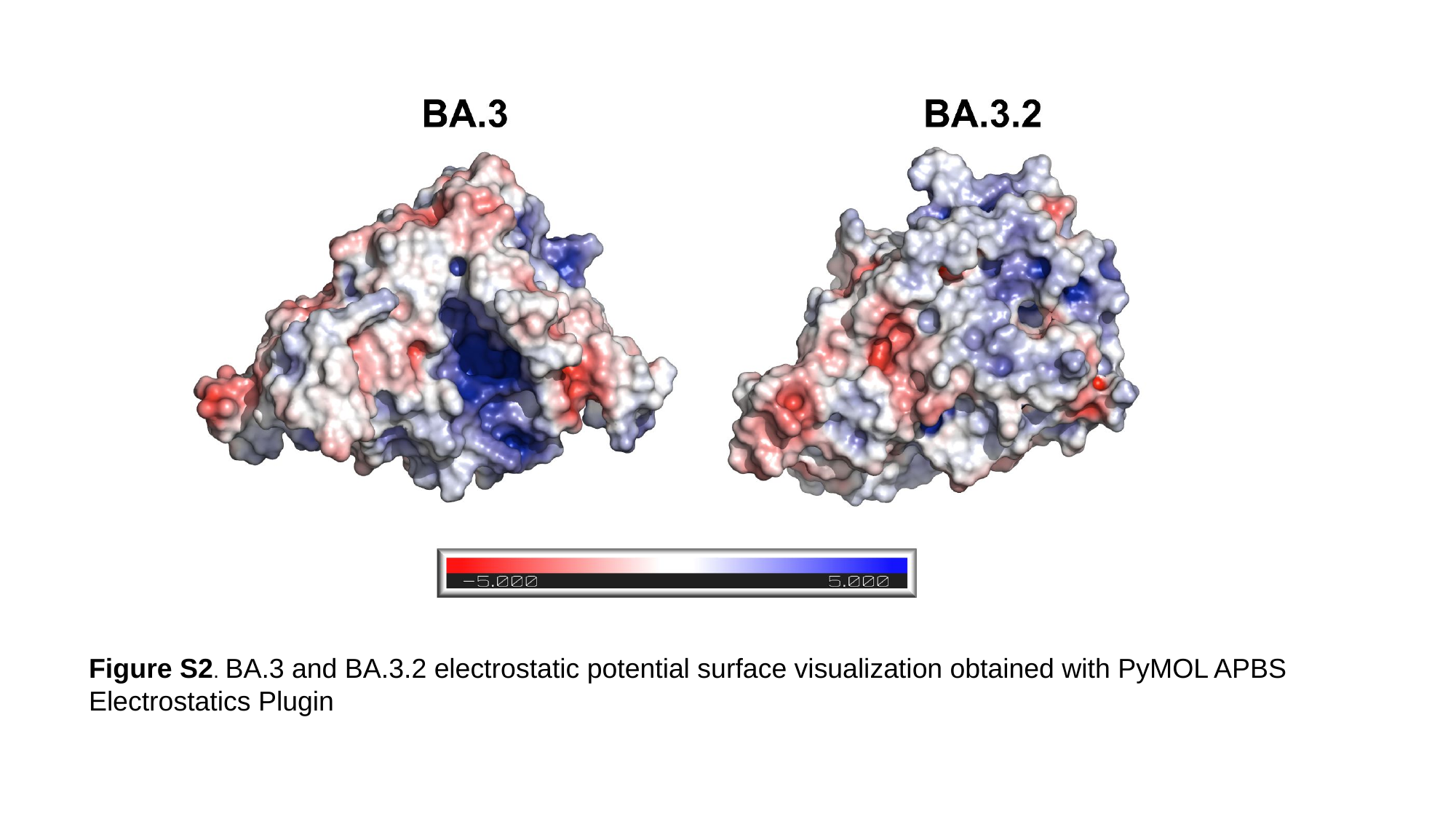

Figure S2. BA.3 and BA.3.2 electrostatic potential surface visualization obtained with PyMOL APBS Electrostatics Plugin

## Slide 4
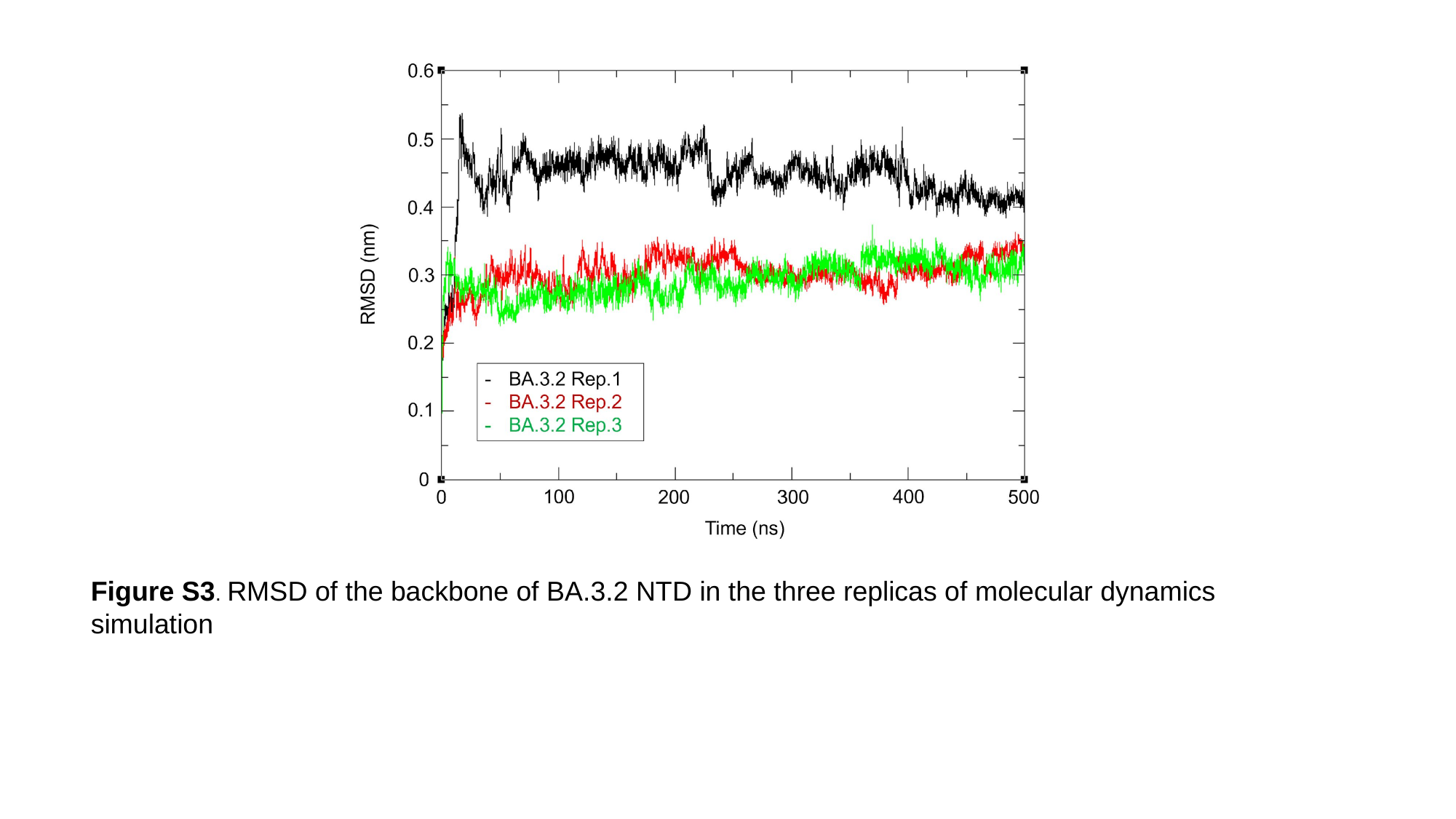

Figure S3. RMSD of the backbone of BA.3.2 NTD in the three replicas of molecular dynamics simulation

## Slide 5
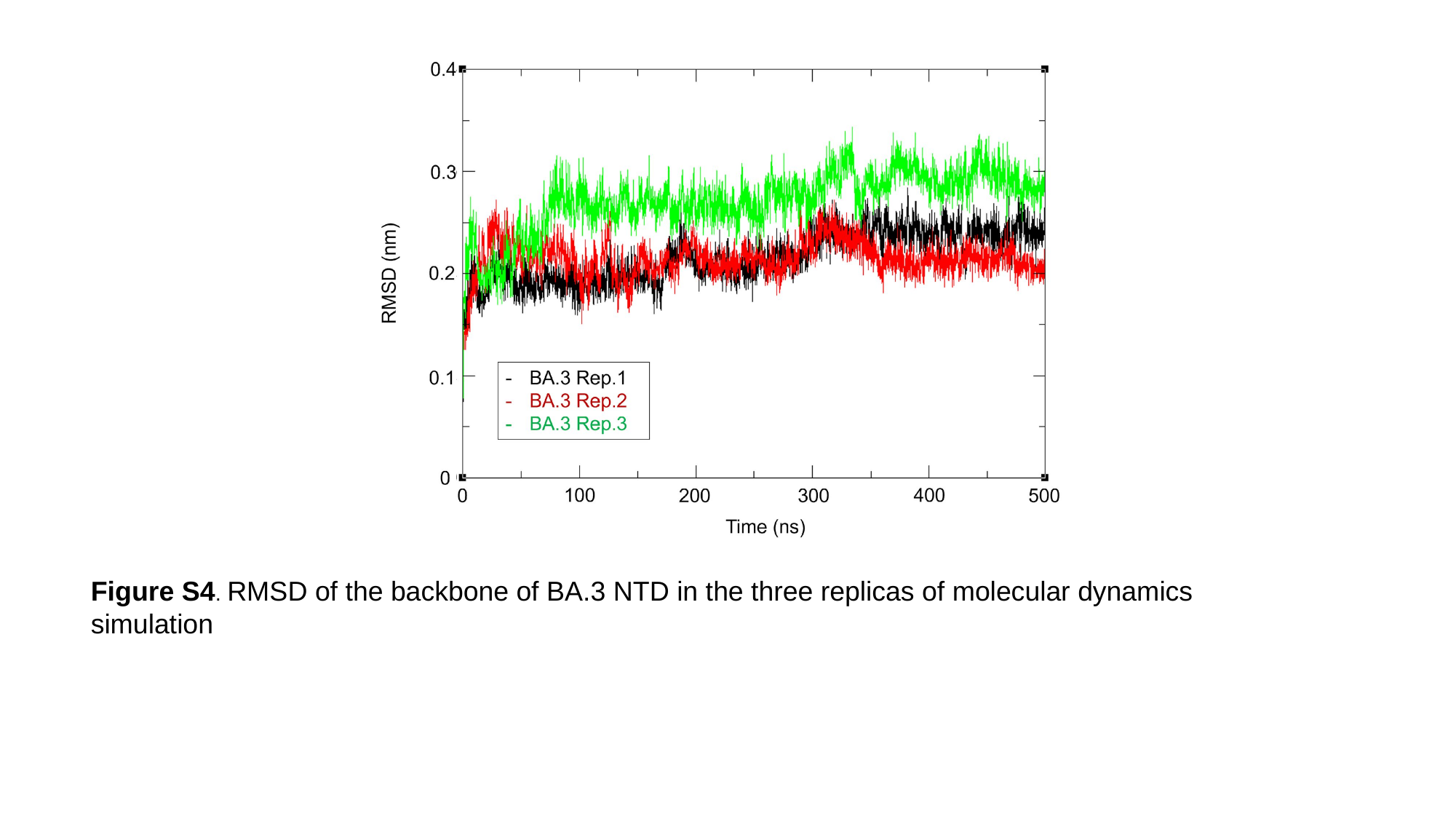

Figure S4. RMSD of the backbone of BA.3 NTD in the three replicas of molecular dynamics simulation

## Slide 6
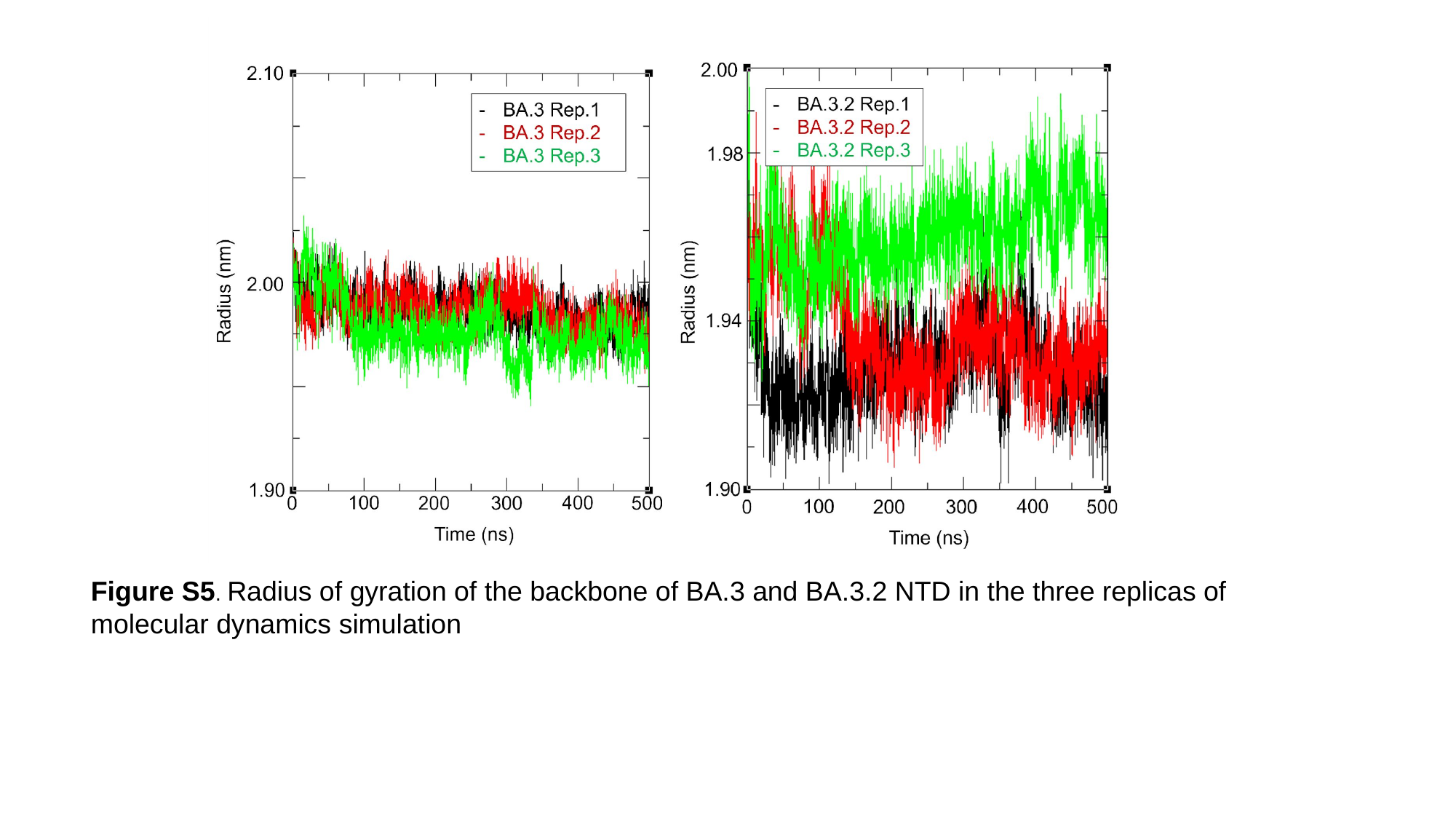

Figure S5. Radius of gyration of the backbone of BA.3 and BA.3.2 NTD in the three replicas of molecular dynamics simulation

## Slide 7
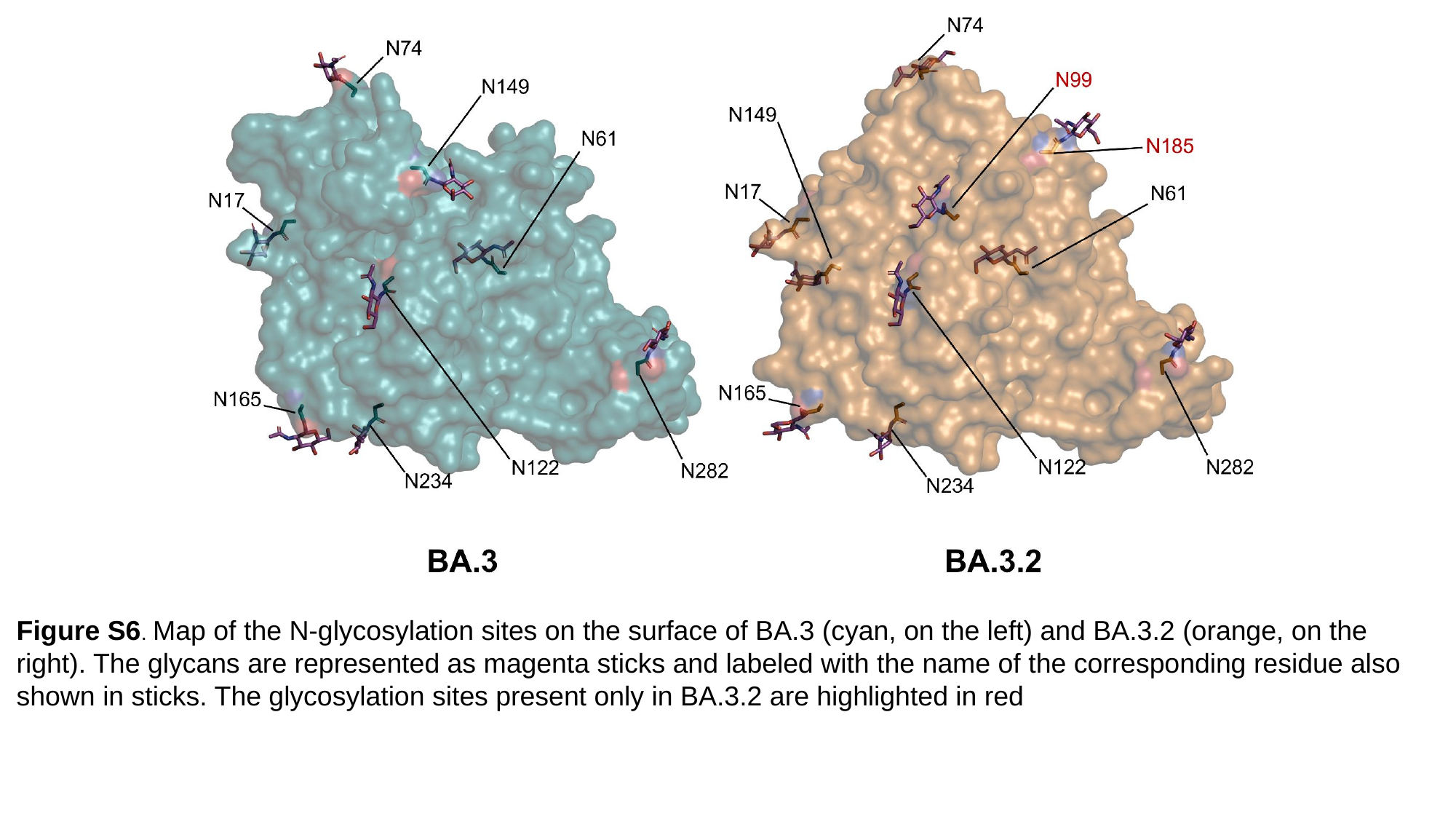

Figure S6. Map of the N-glycosylation sites on the surface of BA.3 (cyan, on the left) and BA.3.2 (orange, on the right). The glycans are represented as magenta sticks and labeled with the name of the corresponding residue also shown in sticks. The glycosylation sites present only in BA.3.2 are highlighted in red

## Slide 8
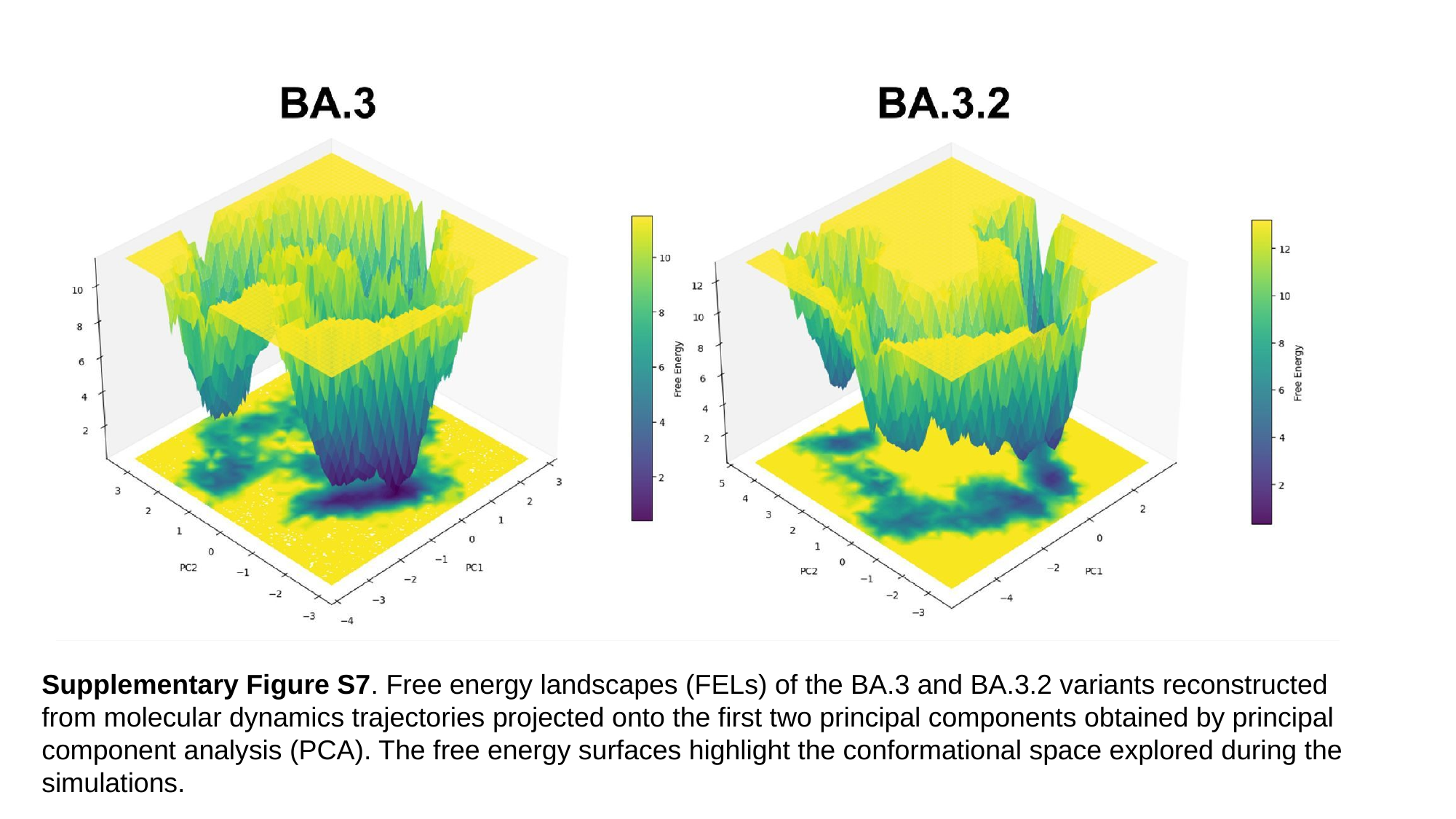

Supplementary Figure S7. Free energy landscapes (FELs) of the BA.3 and BA.3.2 variants reconstructed from molecular dynamics trajectories projected onto the first two principal components obtained by principal component analysis (PCA). The free energy surfaces highlight the conformational space explored during the simulations.
